# Complete Chromosomal Sequences of Two Borrelia miyamotoi Samples Obtained from Ixodes ricinus Eggs in Czechia

**DOI:** 10.1128/MRA.01504-19

**Published:** 2020-04-02

**Authors:** Jan Janeček, Markéta Nováková, Jan Oppelt, Petra Pospíšilová, Anita Cunha, Ana Catarina Silva, Li Dantong, David Šmajs

**Affiliations:** a Department of Experimental Biology, Faculty of Science, Masaryk University, Brno, Czech Republic; b Department of Biology, Faculty of Medicine, Masaryk University, Brno, Czech Republic; c Central European Institute of Technology, Masaryk University, Brno, Czech Republic; d Faculty of Medicine, University of Porto, Porto, Portugal; e Southern Medical University, Guangzhou, China; University of Maryland School of Medicine

## Abstract

Here, we present complete chromosome sequences of Borrelia miyamotoi samples CZ-F1E and CZ-F190E, which were obtained from Ixodes ricinus eggs from Czechia. The chromosome sequences, assembled from Illumina and Sanger sequencing data, had average coverage values of 647× and 3,216×, respectively. They belong to the European genotype, distinct from the Asian and American strains.

## ANNOUNCEMENT

The tick-borne human pathogen Borrelia miyamotoi is distributed in the Holarctic realm (1). Characterization of B. miyamotoi genome sequences, especially those from Europe, is needed since the genome sequences of only two strains from the Netherlands have recently been published (2) and several whole-genome sequences from North America and Asia have been studied (3–7).

In total, 416 engorged females of Ixodes ricinus were manually collected from dogs and cats by their owners in Czechia in 2018 to 2019. Ticks were stored separately in labeled tubes at 26°C and 90% relative humidity. Egg clusters were collected from 364 females promptly after oviposition and were stored in cryovials at −80°C. DNA was extracted from ∼100 eggs per cluster with the DNeasy blood and tissue kit (Qiagen). The presence of B. miyamotoi DNA in 6 of 364 samples was confirmed by amplification of a partial sequence of the *glpQ* gene (8).

The ratio of B. miyamotoi to I. ricinus DNA copies in the six B. miyamotoi-positive samples was determined by nested PCR detection of the *glpQ* gene and I. ricinus *ITS2* spacer (Table 1) in 10-fold serially diluted DNA samples (from nondiluted to 10^−6^ diluted). PCR products from the second amplification step were visualized by 1.5% agarose gel electrophoresis. Two samples, designated CZ-F1E and CZ-F190E, with the highest B. miyamotoi/I. ricinus DNA ratios (i.e., 2:1 and 20:1, respectively) were subjected to whole-genome sequencing. DNA libraries were prepared using the NEBNext Ultra DNA library preparation kit (Illumina) and sequenced on the Illumina platform in paired-end mode with a read length of 150 bp (Novogene, China).

**TABLE 1:** Primer pairs used for PCR amplification

Organism	Target^*a*^	Primer name	Nucleotide sequence (5′ to 3′)	Amplified fragment (bp)	Annealing temp (°C)	Reference or source
*Ixodes ricinus*	*ITS2*	ITS2-outF	CTCTTTGAACGCACATTGCG	∼590^*b*^	48	This study
		ITS2-outR	AGACTGACGGAAGGCTACGA			This study
		ITS2-F	CTGTGTGAGGGTCGGATCAT	∼315^*b*^	60	This study
		ITS2-R	CGTTTCACATCTCCAACGCA			This study
*Borrelia miyamotoi*	*glpQ*	Q1	CACCATTGATCATAGCTCACAG	633	48	8
		Q2	CTGTTGGTGCTTCATTCCAGTC			8
		Q3	GCTAGTGGGTATCTTCCAGAAC	399	48	8
		Q4	CTTGTTGTTTATGCCAGAAGGGT			8
	Gap 1	Gap1-2 F	ACCAAGATTCCTCAATTGCTC	758	58–50	This study
		Gap1-2 R	GATGAAAGCTACTGCTTCAATTGA			This study
	Gap 2	Gap2-2 F	CAATCTTGTCGTTCAGTGTATC	878	58–50	This study
		Gap2-2 R	GAATATAAAACCCTAGCAACAAGC			This study
	Gap 3	Gap3-1 F	CAAGAGTTGTTAGCAGGACTTCA	932	58–50	This study
		Gap3-1 R	AGCAGCAGAACTAATTGTAA			This study
	Gap 4	Gap4-2 F	AGAGAAGGTAGTTGGGGCT	470	58–50	This study
		Gap4-2 R	GTAGCTTTCTCAAGTTGCT			This study

a Amplification steps for *ITS2* and *glpQ* were as follows: 94°C for 1 min; 40 cycles of 94°C for 30 s, with annealing at 48°C (or 60°C) for 30 s and 72°C for 1 min 45 s; and 72°C for 7 min. For gaps 1 to 4, conditions were as follows: 95°C for 3 min; 9 cycles of a touchdown step with denaturation at 94°C for 30 s, with the annealing temperature reduced from 58°C to 50°C, and extension at 72°C for 1 min, followed by 35 cycles of denaturation at 94°C and annealing at 50°C, both for 30 s, and extension at 72°C for 1 min; and a final extension step at 72°C for 5 min.

b The length varies slightly among tick individuals.

The 347,837,033 and 358,142,224 raw reads for CZ-F1E and CZ-F190E, respectively, were adapter and quality (Phred score of <15) trimmed by Cutadapt (v1.15) (9). The preprocessed reads were filtered by mapping to the I. ricinus sequence (GenBank accession number GCA_000973045.2) using BBMap (v37.25), which yielded 268,026,168 and 299,377,980 reads (77.1% and 83.6% of the raw counts) for CZ-F1E and CZ-F190E, respectively (10). The quality of the reads was continuously assessed by FastQC (v0.1.15) (11). The host clean reads were mapped to prokaryotic genomes with Kraken2 (v2.0.7-beta) (12), and the proportions of reads assigned to B. miyamotoi reached 2.5% and 14.4% for CZ-F1E and CZ-F190E, respectively. The fastq sequences were mapped to the B. miyamotoi representative genome LB-2001 assembly using BWA MEM (v0.7.15) (13); mapped reads were extracted using SAMtools (v1.4) and *de novo* assembled by SPAdes (v3.13.0) (14, 15).

For each sample, the five longest scaffolds were manually selected from the assembly graph using Bandage (v0.8.1) (16). The average coverage values were 647× and 3,216× for CZ-F1E and CZ-F190E, respectively.

Gaps between the scaffolds were filled by PCR amplification using custom primers (Table 1) and Sanger sequencing. Annotation was performed using the Prokaryotic Genome Annotation Pipeline (17).

The complete chromosome sequences of 904,129 bp and 904,095 bp for CZ-F1E and CZ-F190E, respectively, with GC contents of 28.7%, contained 810 and 807 predicted protein-coding sequences, respectively, 3 rRNAs, 31 tRNAs, and 28 and 23 pseudogenes, respectively. The differences between the two genomes included 51 single-nucleotide variants, of which 35 nucleotide differences were located in coding regions, resulting in 12 amino acid differences in the predicted proteomes. Future studies characterizing plasmid contents are expected to bring new insights into the genetic diversity of this emerging pathogen.

### Data availability.

The complete chromosome sequences of B. miyamotoi CZ-F1E and CZ-F190E samples have been deposited in the GenBank/DDBJ/EMBL database under accession numbers CP046389 and CP046388, respectively. The corresponding BioProject accession number is PRJNA591086, and the BioSample accession numbers are SAMN13351967 and SAMN13351968. The GenBank accession numbers for sequences obtained by Sanger sequencing are MN990105, MN990106, MN990107, MN990108, MN990109, MN990110, MN990111, and MN990112.
